# Controlled power: how biology manages succinate-driven energy release

**DOI:** 10.1042/BST20211032

**Published:** 2021-12-09

**Authors:** Shona A. Mookerjee, Akos A. Gerencser, Mark A. Watson, Martin D. Brand

**Affiliations:** 1 Department of Biological and Pharmaceutical Sciences, Touro University California College of Pharmacy, Vallejo, CA, U.S.A; 2 Buck Institute for Research on Aging, Novato, CA, U.S.A

**Keywords:** bioenergetics, ischaemia-reperfusion injury, membrane potential, mitochondria, reactive oxygen species, thermodynamics

## Abstract

Oxidation of succinate by mitochondria can generate a higher protonmotive force (pmf) than can oxidation of NADH-linked substrates. Fundamentally, this is because of differences in redox potentials and gearing. Biology adds kinetic constraints that tune the oxidation of NADH and succinate to ensure that the resulting mitochondrial pmf is suitable for meeting cellular needs without triggering pathology. Tuning within an optimal range is used, for example, to shift ATP consumption between different consumers. Conditions that overcome these constraints and allow succinate oxidation to drive pmf too high can cause pathological generation of reactive oxygen species. We discuss the thermodynamic properties that allow succinate oxidation to drive pmf higher than NADH oxidation, and discuss the evidence for kinetic tuning of ATP production and for pathologies resulting from substantial succinate oxidation *in vivo*.

## Introduction

The operation of a biological system is like the operation of a factory: each machine (enzyme) in the factory performs one or more linked tasks. At steady-state, the rate of each reaction in the system is the same. This rate is not, as is sometimes stated, set by the capacity of the slowest machine (the ‘rate-limiting step'). Instead, it is dynamic and responsive; adjustment of the concentrations of the intermediates by the system coordinates the rate of each machine with the rates of all the others linked to it. This coordination helps to avoid the accumulation of too much of one thing or the depletion of another and keeps the factory running smoothly.

Seeing the molecular machines present, and what each one does, is clearly necessary to understanding the system. However, you can only understand how the factory operates when the machines are all running and material is flowing between them. In steady-state, all the machines run at a rate that keeps all intermediates at constant and appropriate levels, and the factory is supported by the supply of raw materials and meets economic demand for the product.

Mitochondrial energetics is an excellent model factory, whose primary function is to transform energy released from substrate oxidation into the transferable currency of ATP. Standard textbook descriptions of this ‘energetic factory' carefully present the architecture of the molecular machines but tend to minimize the factory's moving parts — the thermodynamic and kinetic properties that drive these machines. The goal of this short review is to discuss why and how understanding the moving parts is crucial to understanding the system, and to examine the kinetic tools used by biology to manage the thermodynamic challenges specific to succinate oxidation.

With this lens, we examine why different substrates (NADH and succinate) with different tendencies to donate electrons (redox potential) behave differently from one another. Specifically, why does the less-powerful reductant, succinate, deliver a higher resting (i.e. non-phosphorylating, State IV) mitochondrial protonmotive force (pmf, Δp, the sum of the electrical and pH potentials across the mitochondrial inner membrane) [1]? What biological or pathological implications does high pmf have? Since pmf is linked to both cellular phosphorylation potential (tendency to drive phosphate transfer) and production of reactive oxygen species (ROS: here, superoxide, O_2_^•−^, and hydrogen peroxide, H_2_O_2_), it is an indirect modulator of any process sensitive to ATP/ADP or to ROS. Pmf itself may also drive cell signaling-related responses (such as parkin-dependent mitophagy [2,3])

Why succinate? As an intermediate in the tricarboxylic acid (TCA) cycle, succinate is a small component of normal cellular metabolism. However, there is abundant evidence that succinate accumulates during tissue ischemia [4,5], and that succinate oxidation upon reperfusion causes ROS-dependent damage (e.g. [6]). Succinate accumulation is associated with diverse pathologies, including epilepsy [7], cancer [8–10], and intestinal inflammation [11]. This review illustrates the fundamental bioenergetics underlying the more complicated pathophysiologies of these diseases. In principle, other pathways (such as oxidation of glycerol 3-phosphate and acyl-CoA dehydrogenases within β-oxidation of fatty acids) that feed electrons into the ubiquinone (Q) pool have the same ability as succinate to drive high pmf, but they don't appear to do so *in vivo*, perhaps due to different (and stronger) upstream kinetic constraints.

In the classic complete tricarboxylic acid cycle oxidizing pyruvate, the ratio of NADH to succinate production is fixed at 4 : 1, predicting pmf generation >80% by NADH oxidation. However, by diverting carbon into and out of the cycle, biology can shift reducing equivalent generation entirely to NADH oxidation (e.g. by running pyruvate to citrate and using the citrate for fatty acid synthesis) or strongly to succinate oxidation (e.g. running glutamine to lactate, or oxidizing a pool of succinate generated during prior anoxia). In this way, pmf, phosphorylation potential, and superoxide and hydrogen peroxide production will change with the proportion that each substrate contributes to total oxidation. We discuss how such changes would result from systems oxidizing NADH or succinate.

## Thermodynamic gearing of succinate oxidation drives high mitochondrial pmf

Mitochondrial protonmotive force is established through proton (H^+^) pumping by Complexes I, III, and IV of the electron transport chain to generate the electrical and chemical separation of H^+^ across the mitochondrial inner membrane. The energy to pump these H^+^ derives from electron transfer from an initial donor through the chain of redox centers in the respiratory complexes, ending with reduction in O_2_ to H_2_O. For an electron pair (2e^−^) to enter the respiratory chain, the electron donor must be more reducing than the initial acceptor.

The two electron donor couples generally presented together are NADH_2_/NAD (2e^−^ enter at Complex I) and FADH_2_/FAD (2e^−^ enter at succinate dehydrogenase/Complex II). However, they are not exactly analogous — whereas NADH is an intermediate 2e^−^ carrier between substrates (e.g. pyruvate) and Complex I, FADH_2_/FAD is embedded within succinate dehydrogenase and receives its 2e^−^ directly from succinate oxidation to fumarate — making the FADH_2_/FAD couple more analogous to the FMN moiety of Complex I than to NADH. Therefore, succinate/fumarate is the more appropriate redox couple to compare with NADH_2_/NAD.

The NADH_2_/NAD couple is more reducing than the succinate/fumarate couple, yet succinate oxidation can drive pmf higher than NADH_2_ oxidation can [1]. The thermodynamic ‘gearing ratio' of energy available to work done makes it clear why. The energy available is the redox potential difference (ΔE) between the donor couple and O_2_, multiplied by number of e^−^ transferred (here, [2]). The work done is the number of protons (*n*) pumped across the mitochondrial inner membrane multiplied by the pmf they are pumped against. The higher the ΔE/*n* ratio, the higher the pmf.

**Figure 1.**
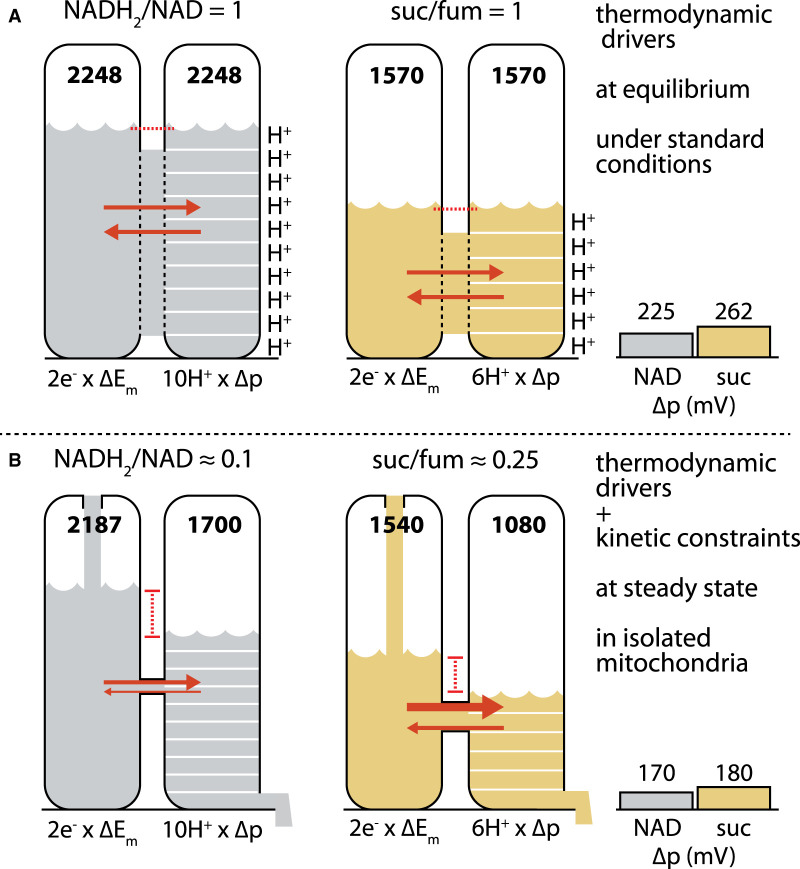
Thermodynamic and kinetic determinants of protonmotive force (pmf, Δp) in (A) isolated mitochondria under standard conditions at equilibrium and (B) liver mitochondria oxidizing excess substrates (succinate or pyruvate/malate) in steady-state resting conditions. (**A**) Under standard equilibrium conditions (50% reduction in redox couples, pH 7.4) at equilibrium, 2ΔE_m_ for NADH_2_ oxidation is 2248 mV, supporting an *n*Δp of 2248 mV (*n* is the gearing: the number of protons pumped as 2e^−^ flow to O_2_). 2ΔE_m_ for succinate oxidation is 1570 mV, supporting an *n*Δp of 1570 mV. Since *n* is 10 for NADH_2_ oxidation and 6 for succinate oxidation, Δp values of 225 mV (from NADH_2_ oxidation) and 262 mV (from succinate oxidation) result. (**B**) Kinetics of supply and demand in liver mitochondria under non-standard conditions and steady-state resting respiration yield lower 2ΔE_h_ than under standard conditions because redox couples are more oxidized: for NADH_2_, 2ΔE_h_ is 2187 mV; for succinate, 2ΔE_h_ is 1540 mV. Net flow between 2ΔE_h_ and *n*Δp requires that *n*Δp < 2ΔE_h_. The diameter of the connecting pipe reflects the restriction of flow between the two pools that contributes to displacement between 2ΔE_h_ and *n*Δp. Succinate oxidation generally operates faster, and closer to equilibrium than NADH_2_ oxidation, shown by a wider pipe diameter and smaller difference in pool levels. These constraints operating on thermodynamic drivers result in Δp of ∼170 mV (NADH_2_) and 180 mV (succinate). Extensive kinetic controls on succinate dehydrogenase/Complex II activity (which alter the diameter of the connecting pipe in this analogy) may serve to keep the pmf supported by succinate oxidation within acceptable limits in cells (see text). Suc/fum: succinate/fumarate. ‘NAD' and ‘suc' in plots at right refer to the NADH_2_/NAD couple and succinate/fumarate couple, respectively.

This situation is illustrated in Figure 1A, where pools of potential energy (‘potential') are represented as liquid in a tank, and energy transduction as flow through a connecting pipe into a linked tank. The left tank in each system (NADH oxidation in gray; succinate in tan) represents redox potential, summed over the two electrons transferred. The right tank represents the potential of the pmf, summed over the *n* protons pumped. For a hypothetical system at equilibrium under standard conditions, flow between connected tanks is unrestricted and each of the donor redox couples is 50% reduced (i.e. the ratio of reduced to oxidized species equals 1). The redox potential (derived from standard reduction potentials measured in electrochemical half cells, e.g. [12]) under these conditions is E_m_, the midpoint potential. Under these conditions at pH 7.4, E_m7.4_ values for NADH_2_/NAD and H_2_O/O_2_ are about −332 mV and +791 mV, respectively (derived from [13]). The ΔE_m_ between them is therefore 1124 mV. Since 2e^−^ are transferred, the total potential is 2 × 1124 = 2248 mV. For succinate/fumarate, E_m_ is about +6 mV, so ΔE_m_ is 785 mV and the total potential is 2 × 785 = 1570 mV.

Because the systems in Figure 1A are at equilibrium, each of the tanks in a paired system has the same potential. In the right tank in each system representing pmf, the potential is divided into units to show its distribution over all *n* protons pumped; for NADH oxidation through Complex I, *n *= 10. Since succinate-derived electrons are not reducing enough to enter at Complex I (electrons, like water, can't be poured uphill), they enter at Complex II and therefore drive proton pumping only from Complexes III and IV, so *n *= 6 [13,14].

The pmf generated by proton pumping can increase to thermodynamic equilibrium with ΔE, as described by the equation

1 $$n_{\mathrm{protons}\;\mathrm{pumped}}\times \mathrm{pmf}=2_{e-\mathrm{transferred}}\times \Delta E$$

which is usefully rearranged to

2 pmf=2ΔE/n

For NADH_2_, pmf solves to (2 × 1124)/10 = 225 mV. For succinate it solves to (2 × 785)/6 = 262 mV. Thus, even though succinate is a weaker reductant than NADH, its available ΔE_m_ is divided by fewer protons pumped (different gearing), so the resulting pmf is higher. A good analogy is pressure (force distributed over surface area). Compared with succinate oxidation, NAD-linked oxidation is ‘heavier' (greater ΔE_m_) but is distributed over a larger ‘area' (H^+^ pumped), resulting in a smaller ‘pressure' (pmf). Consider how lying on a bed of vertical nails is preferable to lying on one nail.

## Biology imposes kinetic constraints to safely contain thermodynamic drivers of mitochondrial pmf

Although standard equilibrium conditions (Figure 1A) clarify the thermodynamics at work here, biology does not operate this way. First, the kinetics of the supply and demand reactions drive the donor couples relatively oxidized, with the mitochondrial NADH_2_/NAD pool ∼10% reduced [15,16], and the succinate/fumarate pool ∼25% reduced [12,17]. This relative oxidation decreases the actual redox potential (E_h_) from the midpoint potential E_m_. In Figure 1B, this is reflected by lower potentials in the left tank of each system relative to Figure 1A. Second, these systems operate in non-equilibrium steady state, indicated by flow into the left tank and out from the right. Potential in the upstream tank must exceed that in the downstream tank, otherwise no energy flow occurs.

Connecting pipes between the left and right tanks are different diameters in the two systems. A smaller pipe diameter represents greater kinetic constraint imposed by the enzyme machinery, holding flow between tanks further from equilibrium. Figure 1B illustrates that, by comparing the electron transfer potential of the NADH_2_/NAD and succinate/fumarate pools to the pmf sustained by each, NADH oxidation operates further from equilibrium than succinate oxidation, further limiting the pmf established by NAD-linked flux. For succinate-linked flux, the pipe is wider, flux is higher (e.g. by more than 2-fold in isolated liver mitochondria, while between 1- and 2-fold in isolated muscle mitochondria) and the potentials therefore are closer to equilibrium [21–23].

What empirical evidence (aside from the redox and pmf measurements that underlie the values in Figure 1B) supports and explains these differences in pipe diameter, i.e. in the magnitude of kinetic constraints at Complex I and Complex II? It is helpful to reiterate that the pipe diameter in Figure 1B represents overall enzyme activity and therefore the kinetics, the abundance, and the regulation of the respective enzymes. The following lines of evidence support slower Complex I flux:

- In isolated mitochondria with saturating substrates, the observed rate of uncoupled NADH-linked respiration (via Complex I) is about half the rate of succinate-linked respiration (via Complex II) [21–23], and about one third when compared at identical, non-zero pmf [1].
- The turnover number (moles substrate transformed per second) for isolated Complex I is not much faster, and may be slower, than Complex II. There are few valid literature comparisons — the turnover numbers of succinate dehydrogenase (SDH) and Complex I have been rarely determined side by side, and experimental conditions (enzyme purification, electron acceptors used) varied between laboratories. Ackrell and colleagues reported a 2-fold higher turnover number for Complex I than for Complex II using the same electron acceptor [24]. Conversely, a 5-fold lower turnover number was found for Complex I in [25].
- Complex II is more abundant than Complex I. In mammalian mitochondria SDH was 1.2–2 fold more abundant than Complex I, based on electron microscopic [26,27], spectrophotometric [24] and electrophoretic methods [28,29].
- Low control of pathway flux is associated with near-equilibrium operation [30]. The control of flux across the respiratory chain has been studied using different approaches of metabolic control analysis. In general, control was distributed across the components of the electron transport chain, and SDH did not have particularly low flux control. Direct control analysis [31–38] indicated similar rate control by Complexes I and II, while calculation from elasticities showed slightly smaller flux control by SDH [1,39,40]. Thus, metabolic control analysis provides little support for particularly near-equilibrium operation of SDH.

Altogether, considering the turnover number and stoichiometry of NADH to succinate oxidation (4 : 1 per complete turn of the TCA cycle oxidizing pyruvate), SDH activity is in excess (parity being a 4-fold lower rate). The relative abundance of Complex II activity may help it operate closer to equilibrium, allowing the generation of high pmf [41].

So, what is the net effect on pmf when these biological kinetic constraints modulate the underlying thermodynamics? As Figure 1B illustrates, in isolated mammalian liver mitochondria, succinate oxidation can sustain a pmf up to ∼180 mV, while NADH_2_ (derived from oxidation of glutamate/malate, pyruvate/malate, and oxoglutarate/malate, in different experiments) oxidation can only sustain a pmf up to 170 mV under the same conditions [1,42]. Thus, isolated mitochondria assayed *in vitro* can sustain a higher pmf on succinate, by ∼10 mV. In mitochondria isolated from cultured cells, the electrical portion (Δψ_m_) differed by ∼15 mV [51,56]. In mitochondria isolated from mammalian skeletal muscle, pmf values differed less, ∼4 mV [57,58]. While all comparable measurements to date show succinate driving pmf higher than NADH_2_, the variation in these differences may at least partially reflect tissue specificity.

The same principle applies when different sites of 2e^−^ entry are compared, demonstrating how predominantly the thermodynamics of the mitochondrial machine predict its operation. Brown et al. found that in isolated liver mitochondria, feeding 2e^−^ at Complex IV (via sulfite oxidation) gave 2ΔE_h_ of 987 mV, ∼65% of the value for succinate oxidation, yet drove a resting pmf of 187 mV, ∼10 mV higher than the 176 mV driven by succinate oxidation [59].

## Kinetic constraints are highly tunable by extensive biological regulation

Biological regulation of Complex II activity *in vivo* is extensive, and why so much regulation exists on this enzyme has remained an open question. A tentative model, drawing from the thermodynamic properties above, is that such regulation exists to rein in this powerful machine in cells, by decreasing its activity (narrowing the pipes) when ATP demand slackens and preventing pmf from being driven too high. Multiple regulatory mechanisms are summarized below.

Complex II/SDH exhibits conformational changes through interaction with many metabolites, including the TCA intermediates succinate, malate and oxaloacetate, ATP, reduced ubiquinone, protons (i.e. low pH), and certain anions [48]. These changes modulate enzyme activity. The mechanism of conformational activation/inhibition remains unknown. However, we speculate that its physiological role is to restrict pmf generation to an optimal range, while retaining the ability to drive high pmf when these restrictions are eased (and also allow pmf generation when NADH oxidation is blocked).

Three endogenous metabolites competitively inhibit SDH: oxaloacetate, itaconate, and malonate. The keto-tautomeric form of oxaloacetate produced by malate dehydrogenase (MDH), is a tightly binding competitive inhibitor [49]. Since MDH is fully reversible, keto-oxaloacetate levels are mostly dependent on mass action; higher NADH_2_/NAD will increase malate/oxaloacetate ratio, decreasing oxaloacetate concentration. This interaction may exist to increase SDH activity when Complex I is unable to oxidize NADH, e.g. at very high pmf [50], though activation by ATP may also explain this observation [51]. Oxaloacetate can also be produced in its enol- form by SDH itself oxidizing malate [61,64,65]. This high-affinity, slow-dissociating tautomer can spontaneously interconvert with its keto- form but too slowly to prevent SDH inactivation; oxaloacetate tautomerase activity rescues SDH activity [66,67]. Itaconate, another competitive inhibitor of SDH, is derived through decarboxylation of citrate by the immune-responsive gene 1 product (IRG1) [56]. Itaconate suppresses respiration in activated macrophages [57,58]. Malonate, mostly thought of as an experimental inhibitor, has been detected in brain [59]. Endogenous malonate is also implied by the presence of a malonyl-CoA synthase (ACSF3) [60].

SDH is also subject to posttranslational phosphorylation and succinylation. Mitochondrial Fgr tyrosine kinase phosphorylates SDH on the A subunit and activates the enzyme [61]. Phosphorylation is triggered by H_2_O_2_, but not O_2_^•−^. A corresponding matrix-localized phosphatase, PTPMT1, is reported [62]. Succinylation may be non-enzymatic when succinyl-CoA is high, but succinyl-CoA synthases such as α-KGDH may also succinylate SDH and other proteins, at least in the nucleus [63]. Desuccinylation can occur through SIRT5 activity in the mitochondrial matrix [64].

Complex I also undergoes reversible conformation changes (activation/deactivation A/D transition). One putative function of deactivation is to mitigate reperfusion injury during anoxia [65]. In tissues, there is an equilibrium between A- and D-forms, influenced by a range of factors: SH-oxidation, divalent cations and fatty acids [65]. Furthermore, multiple phosphorylation sites have been proposed; possibly phosphorylation increases Complex I activity by bolstering assembly [66].

## Portioning of the cellular ATP budget is tied to pmf

How cells consume ATP is partly dependent on cellular phosphorylation potential [67]. This form of potential energy is close to equilibrium with pmf, with higher pmf sustaining a higher phosphorylation potential and ATP/ADP ratio. The pmf therefore determines the supply side of the ATP/ADP ratio, a key piece of metabolic information ‘read' by every ATP-binding protein in the cell [13,68]. As pmf changes, the ATP/ADP ratio also changes, which tunes consumption by different energetic processes. This reflects a sensible underlying biological goal: to maintain essential functions while making less-essential functions dependent on the available potential. This was demonstrated by titrating pmf with mitochondrial inhibitors, revealing that as pmf declines, protein synthesis decreases the most, while ion cycling decreases the least [69,70].

As an analogy, when your paycheck is high, you might spend more on luxuries than when times are lean. However, your rent stays about the same. Some categories of your spending (luxuries) are more dependent on your income than others (rent). In biology, the implication is that sustained high pmf through oxidation of succinate drives proportionally more protein synthesis. Pmf is therefore not just an intermediate to be charged and discharged; in the light of an ATP consumption hierarchy, pmf becomes an important director of cellular activity.

As an aside, a common oversimplification is that high pmf is ‘functional', and low pmf is ‘dysfunctional'. A model that better fits the available data is that both extremes carry the risk of dysfunction; too low a pmf leaves a cell under-powered and unable to run essential functions, whereas too high a pmf drives high rates of superoxide production and leads to damage and pathology (section 6). What defines ‘extreme' remains to be determined but it likely cell type-specific. Plausibly, pmf values between these extremes are neither strictly functional nor dysfunctional; the biological outcome depends on how pmf supports cellular functions and viability in the environment occupied by the biological system (defined as narrowly or as broadly as appropriate). For example, in both neurons and pancreatic β-cells the pmf can change by up to 50 mV as a part of apparently normal function [71,72]

## Mitochondrial superoxide and hydrogen peroxide production rises with pmf

As well as driving high ATP/ADP, high pmf also slows electron flow, reducing redox centers, which drives faster mitochondrial production of superoxide and hydrogen peroxide. Succinate oxidation can therefore drive faster mitochondrial O_2_^•−^/H_2_O_2_ production than NADH oxidation [73–75]. Like water overflowing a clogged sink, mitochondrial O_2_^•−^/H_2_O_2_ production rate increases at high pmf when the ubiquinone pool is highly reduced (sink full of water) and single electrons more readily reduce O_2_ to form O_2_^•−^ (water pooling on the floor). In isolated mitochondria O_2_^•−^/H_2_O_2_ production increases non-linearly with respect to pmf; between pmf values of ∼180 (achieved by NADH oxidation) and 220 mV (achieved by succinate oxidation) O_2_^•−^/H_2_O_2_ production can increase 5- to 10-fold [76–79].

Considering the framework presented above, isolated mitochondria can be exposed to conditions (such as exclusive oxidation of abundant succinate) that bypass the biological constraints that would presumably be present *in vivo* to prevent damage and pathology. Are there conditions in cells and *in vivo* that approximate the unconstrained state achievable *in vitro*, and are they pathological?

First, can high pmf drive O_2_^•−^/H_2_O_2_ production when mitochondria are within cells or *in vivo*? Yes — mitochondria are a major contributor to cellular H_2_O_2_ production, with the majority emanating from site I_Q_ of Complex I and site III_Qo_ of Complex III, the sites are routinely driven in isolated mitochondria by the oxidation of excess added succinate [80–83]. O_2_^•−^/H_2_O_2_ production by sites I_Q_ and III_Qo_ is not restricted to cell models; substantial pathological effects of H_2_O_2_ production from sites I_Q_ and III_Qo_ can also be demonstrated *in vivo* when endogenous mitochondrial superoxide dismutase is knocked out [84]. Under conditions of low succinate and high pmf, Complex II is also a significant contributor to ROS production, which could be biologically relevant [85,93], but would contribute less to overall ROS production as succinate accumulates [85].

Second, can succinate levels rise high enough *in vivo* that succinate oxidation could drive high rates of O_2_^•−^/H_2_O_2_ production? The answer is yes, with the best-studied model being ischemia/reperfusion (I/R) injury [86]. Estimated intramitochondrial succinate levels in normal rat skeletal muscle are 200–300 µM [80], in the range of the *K*_M_ of SDH for succinate (100–400 µM [58,87] in mammalian mitochondria). In contrast, succinate levels can accumulate 3- to 20-fold during ischemia [6]. Reperfusion drives rapid oxidation of the accumulated succinate, high pmf, a reduced ubiquinone pool, and high rates of O_2_^•−^/H_2_O_2_ production. Succinate accumulation appears to amplify the HIF-1-dependent hypoxic response [88], suggesting that succinate accumulation is a common feature of hypoxia more broadly.

Two plausible models explain why succinate accumulates under ischemia. The absence of sufficient O_2_ can lead cellular reductants to drive SDH in reverse, pulling fumarate from different sources and reducing it to succinate. Alternatively, succinate may accumulate through forward SDH activity, supported partially by aminotransferase anaplerosis [4,6].

Having evidence that O_2_^•−^/H_2_O_2_ production from sites I_Q_ and III_Qo_ occurs *in vivo*, and that succinate can accumulate *in vivo*, it needs to be demonstrated that oxidation of accumulated succinate is associated with high rates of O_2_^•−^/H_2_O_2_ production from these sites. Strong evidence comes from experiments showing that tissue damage due to I/R is attenuated when different elements required for succinate oxidation-associated O_2_^•−^/H_2_O_2_ production are disrupted, by inhibiting succinate oxidation [89,90], dissipating pmf (and oxidizing the QH_2_/Q pool) using chemical uncouplers [91,92], inhibiting electron transport through site I_Q_ [93,94], or by suppressing O_2_^•−^/H_2_O_2_ production at site I_Q_ using SIQELs, which impedes electron leak to O_2_^•−^/H_2_O_2_ but not electron flux to H_2_O [95,96]. Supporting the idea that O_2_^•−^/H_2_O_2_ derived from site I_Q_ has broad biological effects, S1QELs modulate many different physiological and pathological conditions *in vivo* [74,95].

## Conclusion

Oxidation of succinate supports a higher pmf than oxidation of NADH. This is not because of some specific molecular property of succinate or NADH, but because of the thermodynamics (redox potentials), gearing (numbers of protons pumped) and kinetics of the components of the pathways by which energy from NADH and succinate oxidation is captured as pmf. In the absence of kinetic constraints, thermodynamics enables succinate to drive a pmf high enough to cause significant biological damage. This may explain the extensive regulation of SDH: biology has responded to the thermodynamic hazard created by succinate oxidation by tightly controlling SDH activity to maintain pmf within a range that allows optimal ATP production and signaling (by ATP/ADP, O_2_^•−^/H_2_O_2_, metabolite levels) but minimizes oxidative damage by O_2_^•−^/H_2_O_2_. If these controls are compromised, pathology can follow.

## Perspectives

- We discuss the importance of thermodynamic and kinetic properties in explaining the mitochondrial protonmotive forces that result when different substrates are oxidized.
- Current thinking is complicated when the molecular properties of different substrates and substrate oxidation pathways are emphasized; models of mitochondrial function can be unified and simplified by considering these thermodynamic and kinetic properties.
- Predicting how mitochondrial oxidation of succinate may contribute to physiology and to pathology can be simplified by applying these considerations.

## Abbreviations

MDH: malate dehydrogenase
ROS: reactive oxygen species
SDH: succinate dehydrogenase
TCA: tricarboxylic acid
